# In Vivo Methods to Study Protein–Protein Interactions as Key Players in *Mycobacterium Tuberculosis* Virulence

**DOI:** 10.3390/pathogens8040173

**Published:** 2019-10-01

**Authors:** Romain Veyron-Churlet, Camille Locht

**Affiliations:** Institut Pasteur de Lille, CHU Lille, CNRS, Inserm, Université de Lille, U1019 - UMR 8204 - CIIL - Center for Infection and Immunity of Lille, F-59000 Lille, France; camille.locht@pasteur-lille.fr

**Keywords:** tuberculosis, *Mycobacterium*, protein–protein interactions, virulence

## Abstract

Studies on protein–protein interactions (PPI) can be helpful for the annotation of unknown protein functions and for the understanding of cellular processes, such as specific virulence mechanisms developed by bacterial pathogens. In that context, several methods have been extensively used in recent years for the characterization of *Mycobacterium tuberculosis* PPI to further decipher tuberculosis (TB) pathogenesis. This review aims at compiling the most striking results based on in vivo methods (yeast and bacterial two-hybrid systems, protein complementation assays) for the specific study of PPI in mycobacteria. Moreover, newly developed methods, such as in-cell native mass resonance and proximity-dependent biotinylation identification, will have a deep impact on future mycobacterial research, as they are able to perform dynamic (transient interactions) and integrative (multiprotein complexes) analyses.

## 1. Introduction

*Mycobacterium tuberculosis* (*Mtb*) is the main causative agent of human tuberculosis (TB), which is the leading global cause of death due to a single infectious agent. In 2017, TB killed an estimated 1.6 million people, according to the World Health Organization. In addition, there is an alarming increase in multi-drug resistant TB cases (0.6 million cases in 2017). Therefore, actions to fight TB have to be urgently taken and understanding the mechanisms underpinning mycobacterial virulence, such as signaling pathways [1], transport across the mycobacterial cell wall [2] or lipid metabolism [3], may be useful to tackle TB.

Proteins perform various key roles in bacteria (enzymatic reactions, transport, DNA replication, etc.), either alone or in association with other partners as part of stable or dynamic complexes. Thus, elucidating the role of individual proteins is essential to understand the physiology of bacteria, including *Mtb*. Moreover, deciphering protein–protein interactions (PPI) is crucial not only to understand bacterial physiology but also to elucidate host–pathogen interactions [4]. In addition, studying PPI may facilitate the discovery of unknown protein functions by the ‘guilty by association’ principle, implying that the partner(s) of a protein with unknown function may provide valuable information about the function of that protein [5]. This may potentially lead to the identification of new antibacterial drug targets.

The aim of this review is to provide an overview of the in vivo methods used for the characterization of PPI in mycobacteria and to highlight the pros and cons for each method. Several examples will illustrate how these studies contributed to decipher the mycobacterial interactome, providing worthy insights into *Mtb* virulence mechanisms [6]. This review will focus only on in vivo methods, and in vitro methods, such as co-precipitation, surface plasma resonance or isothermal titration calorimetry, will not be discussed here.

## 2. Yeast Two-Hybrid (Y2H) System

### 2.1. Principle

**Figure 1:**
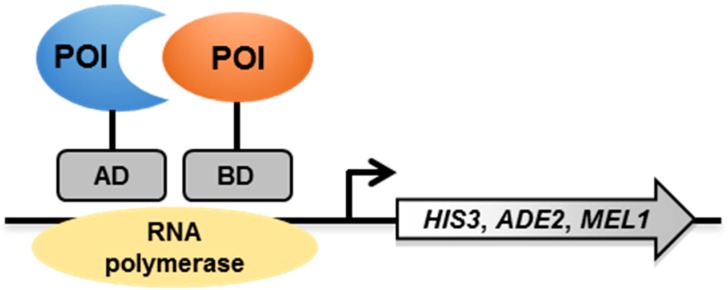
Schematic representation of the yeast two-hybrid (Y2H) system. POI—protein of interest; AD—activating domain; BD—binding domain.

The Y2H system is based on the reconstitution of an active transcriptional activator (TA) in yeast (e.g., GAL4 or LexA) [7]. The proteins of interest (POIs) are produced as chimeric proteins with the DNA-binding domain (BD) or the activating domain (AD) of the TA (Figure 1). If the two proteins under investigation interact, the BD and AD are close enough to each other to allow the transcription of reporter genes, usually auxotrophic markers (*HIS3*, *ADE2* and *MEL1*) or *lacZ*, which in turn allows yeast colonies to grow on selective media or to change color on colorimetric media.

### 2.2. The Y2H System to Study Mycobacterial PPI

#### 2.2.1. Signaling Pathways

Sigma factors are subunits of the RNA polymerase complex required for transcriptional initiation of specific sets of genes. As rapid adaptation is key to the success for bacterial pathogens, sigma factors play a critical role in *Mtb* physiology and virulence [8]. Among the dozens of sigma factors in *Mtb*, SigA, also called RpoV, is essential for growth and is involved in the transcription of housekeeping genes [9]. To study mycobacterial PPI involving SigA, the Y2H system was used to screen a *Mtb* H37Rv library, which led to the identification of the transcriptional regulator WhiB3 as an interactor of SigA/RpoV [10]. In addition, a single amino acid change in SigA/RpoV (R515H) was sufficient to abolish its interaction with WhiB3 in the Y2H system [10]. Another transcriptional regulator, WhiB1, was shown to interact with the alpha-glucan branching enzyme GlgB [11]. SigF is the general stress response sigma factor of *Mtb* and is responsible for the regulation of genes involved in cell wall protein synthesis and in the survival of the bacilli in the host [12,13]. The Y2H system was also used to study the interactions between anti-anti-sigma factor, anti-sigma factor RsbW, and sigma factor SigF [14]. 

In addition to sigma factors, the *Mtb* genome encodes a dozen two-component systems (TCS), allowing gene expression to adapt in response to a wide variety of signals. Some of these TCS were shown to be involved in the regulation of virulence [15]. The Kdp signal transduction pathway appears to be the primary response mechanism to osmotic stress, which is mediated by differences in the potassium concentrations within the bacteria. The N-terminal sensing module of the histidine kinase KdpD interacts with a portion thought to be cytosolic of two membrane lipoproteins, LprF and LprJ, to modulate *kpd* expression [16]. Another study assessed pairwise interactions in the Y2H system between histidine kinases and response regulators of all the mycobacterial TCS in order to assess crosstalks between the different TCS [17].

The *Mtb* genome also encodes eleven serine/threonine protein kinases (STPK), from PknA to PknL. As the *Mtb* phosphoproteome includes hundreds of Ser- and Thr-phosphorylated proteins that participate in many aspects of *Mtb* biology (signal transduction, cell wall synthesis, pathogenesis, etc.), STPK are critical for the regulation of *Mtb* physiology [18]. In an extensive study, 492 STPK interactants were identified by a *Mtb* proteome microarray [19]. To confirm the in vitro screening, the Y2H system was further used to assess the interactions between 75 randomly-selected interactants with PknB, PknD, PknG, and PknH. However, only 52% (39 out 75) of the STPK interactants could be confirmed by the Y2H system, which may be due to the fact that PknB, PknD, and PknH were tested without their membrane domain, as this could have been detrimental in the Y2H system [19].

#### 2.2.2. *Mtb* Cell Division

As for other living organisms, mycobacterial cell growth and division needs to be tightly organized and regulated [20]. In particular, divisome assembly depends on the proper localization of FtsZ in order to form the Z-ring structure [20]. Thus, a Y2H screening was performed using *Mtb* FtsZ as a bait, which led to the identification of SepF (Rv2147c), an essential protein of the division machinery in mycobacteria [21]. 

Reactivation of dormant *Mtb* requires the resuscitation-promoting factors (Rpf), which are peptidoglycan–hydrolyzing enzymes [22]. The Y2H system was used to identify a RpfB and RpfE interactant, named RipA for Rpf-interacting protein A [23]. Additional work performed with RipA as a bait in the Y2H system further identified the protein PBP1/PonA1 as a new partner, potentially modulating the RipA–RpfB cell wall degradation activity [24].

#### 2.2.3. *Mtb* Cell Wall Composition

Mycolic acids are essential lipid components of the mycolic acid–arabinogalactan–peptidoglycan complex (MAPc) in the *Mtb* cell wall and they contribute directly to the pathogenicity of *Mtb* [25]. The Y2H system was extensively used to demonstrate that the discrete enzymes of the fatty acid synthase-II (FAS-II) system interact with each other during mycolic acid biosynthesis, suggesting the existence of specialized and interconnected protein complexes [26,27,28].

Another study using the Y2H system showed that Rv2623, a universal stress-response protein, and Rv1747, a putative ATP-binding cassette transporter, interact with each other to regulate mycobacterial growth by potentially impeding Rv1747 function as a phosphatidylinositol mannoside (PIM) transporter [29]. PIM are immunologically active lipids that can modulate the host immune response [30,31].

#### 2.2.4. Secretion of *Mtb* Virulence Factors

ESAT-6 and CFP-10 are both secreted antigens, which play a key role in *Mtb* virulence [32,33]. The Y2H system helped to demonstrate that EccCa1, EccCb1, and EccD1, which are components of the type VII secretion system ESX-1 [34,35], are required for ESAT-6/CFP-10 secretion [36]. In addition, a single amino acid change in the C-terminal region of CFP-10 was enough to abolish the CFP-10/EccCb1 interaction in the Y2H system, and to prevent secretion of the ESAT-6/CFP-10 complex [37]. Similar to CFP-10, the C-terminal region of EspC, another ESX-1 substrate, was shown to interact with Rv3868, a cytosolic ATPase, by a Y2H approach [38].

#### 2.2.5. Regulation of Mycobacterial Protease Activity

Mycobacterial proteases play critical roles in pathogenesis [39]. For instance, the site-2 protease Rip1 (Rv2869c) is a major virulence determinant in *Mtb* [40,41]. A Y2H screening performed using the Rip1 PDZ domain against a *Mtb* library led to the identification of PDZ-interacting protease regulators 1 and 2 (Ppr1 and Ppr2, corresponding to Rv3333c and Rv3439c, respectively) and these interactions are thought to prevent nonspecific activation of the Rip1 pathway [42].

### 2.3. Pros and Cons

The Y2H system allows direct assessment of pairwise interactions between partners in an in vivo context. However, as the readout is based on transcription factors active in the nucleus of the yeast cell, the Y2H system requires nuclear translocation of the proteins under study. Thus, membrane-associated proteins are difficult if not impossible to study in this system (Table 1). Furthermore, only two (or three in the case of a Y3H system) partners can be studied at a time. In addition, the Y2H system is not suitable for the study of PPI in their natural cellular context, and specific mycobacterial post-translational modifications (PTM) or cofactors may be lacking in yeast (Table 1).

## 3. Bacterial Adenylate Cyclase-Based Two-Hybrid (BACTH) System

### 3.1. Principle

**Figure 2:**
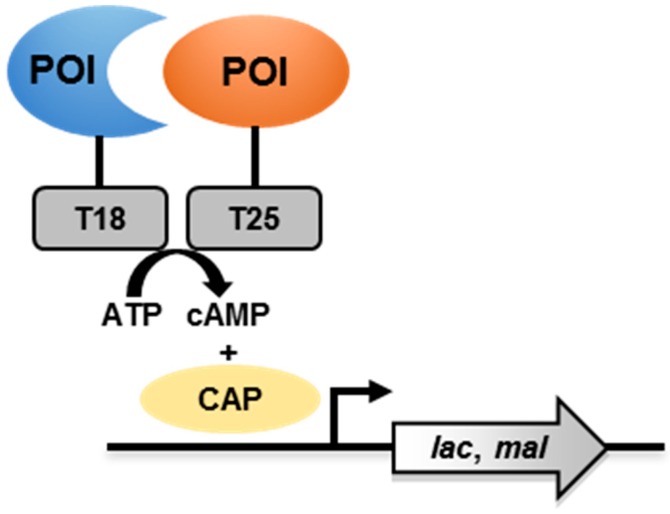
Schematic representation of the bacterial adenylate cyclase-based two-hybrid (BACTH) system. ATP—adenosine triphosphate; cAMP—cyclic adenosine monophosphate; CAP— catabolite activator protein.

The BACTH system is based on the interaction-mediated reconstitution of an active *Bordetella pertussis* adenylate cyclase (CyaA) in *Escherichia coli* [43,44,45]. POIs are genetically fused to the N-terminal or C-terminal ends of the subunits T18 or T25 of CyaA (Figure 2). The enzyme is inactive when T18 and T25 are physically separated. When the POIs interact, the proximity of T18 and T25 allows the generation of cyclic adenosine monophosphate (cAMP), which then binds to the catabolite activator protein (CAP). This cAMP/CAP complex then activates the transcription of reporter genes (*lac* and *mal* operons). As *lac* and *mal* operons are involved in lactose and maltose catabolism, respectively, this allows *E. coli* to grow on media on which lactose or maltose is the unique carbon source.

### 3.2. The BACTH System to Study Mycobacterial PPI

#### 3.2.1. Signaling Pathways

*Mtb* SigE plays an important role in the intracellular life of mycobacteria and regulates the expression of several genes that are important for maintaining the integrity of the cell envelope during stress, particularly during macrophage infection, since SigE is required to arrest phagosome maturation [46,47]. SigE interacts with the anti-sigma factor RseA in the BACTH system, and using this system residues C70 and C73 of RseA have been shown to be required for full interaction, which prevents the transcription of genes that are controlled by SigE [48].

The BACTH system was also used to study the interactions between components of TCS, such as the C-terminal domain of the response regulator MtrA and the histidine kinase MtrB [49]. Although the environmental signals sensed by MtrA/B are unknown, these TCS are essential for mycobacterial growth. On the other hand, overexpression of *mtrA* was shown to impede in vivo proliferation of *Mtb* [50,51]. In another study, the sensor kinase KdpD was found to interact in the BACTH system with the membrane peptide KpdF, potentially altering KdpABC transporter function [52]. In the same study, a screening was performed using KdpF as a bait against a *Mtb* H37Rv DNA library, which led to the identification of MmpL7 and MmpL10 as interactors [52]. These proteins are members of the MmpL protein family involved in lipid and iron transport in mycobacteria [53,54]. It was further shown that KdpF also interacts with the nitrosative stress detoxification proteins NarI and NarK2, as well as with a protein highly induced upon nitrosative stress, Rv2617c [55]. This PPI network suggests that the KdpF peptide could promote the degradation of these partners involved in nitrosative stress, leading to decreased intracellular multiplication of the mycobacteria [55].

#### 3.2.2. Cell Division

The BACTH system was also used to characterize the mycobacterial cell division. It allowed the identification of interactions between FtsW, FtsZ, and PbpB [56]. Another study demonstrated that FtsZ is able to interact with ClpX, the substrate-recognition domain of the ClpXP protease, potentially modulating Z-ring structure formation and negatively regulating FtsZ polymerization [57]. FtsZ also interacts with CrgA (Rv0011c), a protein that possibly facilitates septum formation [58]. Another study showed that the membrane protein CwsA (Rv0008c) interacts with CrgA and Wag31, both involved in mycobacterial peptidoglycan biosynthesis [59]. Together these studies highlight the value of the BACTH system to characterize the mycobacterial divisome [60].

#### 3.2.3. *Mtb* Cell Wall Composition

A BACTH screening using as a bait KasA, a component of FAS-II system, revealed that KasA interacts with PpsB and PpsD, which are two enzymes involved in the biosynthesis of lipid phthiocerol dimycocerosate (PDIM). This suggests a possible transfer of lipids between the FAS-II system and the PDIM biosynthetic pathways [61], highlighting the importance of PPI in the course of mycobacterial cell wall biosynthesis. Similar to mycolic acids, PDIM are involved in mycobacterial virulence [62,63].

EccA1 is an ATPase and belongs to ESX-I, the mycobacterial type VII secretion system [34]. It was shown that the *Mycobacterium marinum* EccA1 activity is required for optimal mycolic acid biosynthesis, probably through its interaction with FAS-II components (KasA and KasB), the mycolic acid condensase Pks13, and potentially with the mycolic acid methyltransferase MmaA4 [64]. In addition, EchA6, a putative enoyl-CoA hydratase, also interacts with several members of the FAS-II system (KasA and InhA), suggesting a possible role in feeding FAS-II with long-chain fatty acids [65].

The BACTH system was also used to detect interactions between the transporter-like Rv3789 and the galactosyltransferase Glft1, involved in arabinogalactan biosynthesis, another component of the mycobacterial MAPc [66].

Recently, a *Mtb* genome-wide screening using MmpL3 as a bait in the BACTH system identified several interactants related to mycolic acid biosynthesis (MmpL11 and Rv0228 = TmaT), peptidoglycanbiosynthesis (Rv3909, Rv3910 and Rv1337), glycolipid biosynthesis (Rv0227c, Rv0236c = AftD and Rv1457c), and cell division (CrgA) [67].

#### 3.2.4. *Mtb* Virulence Factors

The BACTH system was used to search for partners of the virulence-associated factor Erp, which is required for optimal multiplication of *Mtb* in murine bone marrow-derived macrophages and in vivo in mice [68]. This led to the identification of two putative membrane proteins, Rv1417 and Rv2617c [69], the functions of which remain to be established.

MgtC is a virulence factor that participates to the adaptation of mycobacteria to magnesium deprivation [70]. The BACTH system was used to assess the interactions between MgtC from *Mtb* and a MgtR peptide from *Salmonella typhimurium* [71], known to promote MgtC degradation in *Salmonella* [72]. Thus, the BACTH system is also useful to evaluate the anti-virulence activity of peptides (or proteins).

HbhA is a surface-exposed adhesin that is involved in the binding of mycobacteria to non-phagocytic cells, a necessary process for *Mtb* dissemination [73], and in the formation of intracellular lipid inclusions [74]. The BACTH system was used to demonstrate that HbhA interacts with Rv0613c and MmpL14 [75]. In addition, deletion of the orthologous gene of *rv0613c* in *Mycobacterium smegmatis* prevents cell-surface exposure of HbhA [75], illustrating that the BACTH system can be helpful to start deciphering novel protein secretion mechanisms.

A three-hybrid system was developed in *E. coli* and helped to confirm the interactions between ESAT-6, CFP-10, and EccCb1 [76], as previously described with individual binary interactions identified in the Y2H system [36,37].

#### 3.2.5. High-Throughput Screening Applied to BACTH

The BACTH system has mostly been used to study pairwise interactions between a limited number of proteins. However, a global *Mtb* PPI network was also studied using the BACTH system. By using the nearly complete *Mtb* gene sets, it led to the identification of more than 8000 interactions involving 2907 mycobacterial proteins [77]. All these potential interactions now require further validation and characterization using complementary approaches.

### 3.3. Pros and Cons

Like the Y2H system, the BACTH system also permits to test the direct interactions of pairwise partners in an in vivo environment and is limited to detect binary interactions (or potentially ternary interactions in the case of the bacterial three-hybrid system). However, unlike the Y2H system, membrane-associated proteins can be studied in the BACTH system, as long as T18 and T25 reside in the cytoplasmic compartment of the bacteria. The bacterial cellular context is partially maintained but it lacks the specificity of the mycobacterial cell wall organization (Table 1). Finally, some bacterial PTM and cofactors may be present in *E. coli*, however, all specific mycobacterial PTM and cofactors are absent (Table 1).

## 4. Methods Developed for Use with Live Mycobacteria

### 4.1. The Mycobacterial Protein Fragment Complementation (M-PFC)

**Figure 3:**
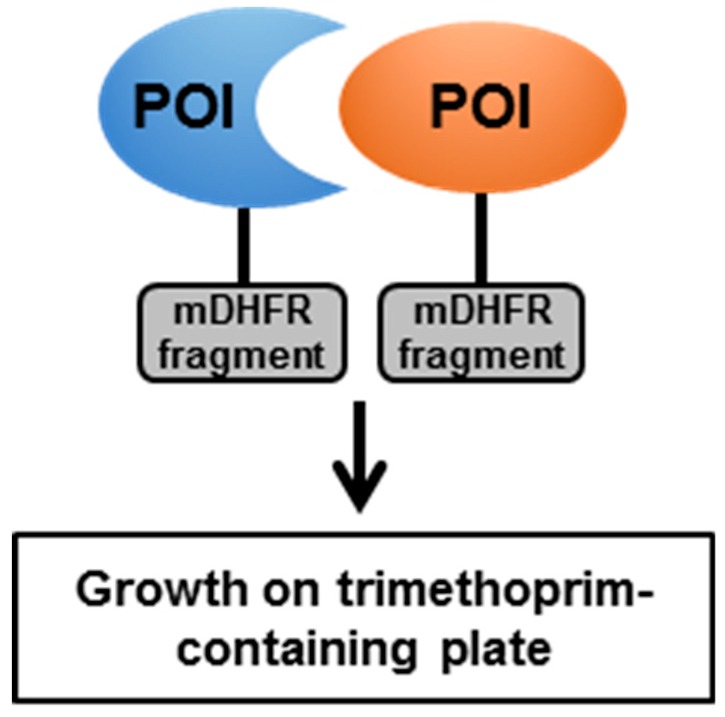
Schematic representation of the mycobacterial protein fragment complementation (M-PFC) technology. mDHFR—murine dihydrofolate reductase.

The Y2H and BACTH systems have their limitations, as the identified interactions do not necessarily occur in their natural environment. In addition, neither system can take care of the specific mycobacterial cell wall organization, and some of the specific PTM and cofactors (Table 1). Hence, systems to directly assess PPI in a mycobacterial environment have been developed. The mycobacterial protein fragment complementation (M-PFC) technology relies on the functional reconstitution of a murine dihydrofolate reductase (mDHFR) in *M. smegmatis* [78]. The POIs are fused to complementary fragments of mDHFR (Figure 3). If the POIs interact, the reconstitution of an active mDHFR confers resistance to the antibiotic trimethoprim. This system was validated by confirming the interactions between ESAT-6 and CFP-10, membrane-associated DosS and cytosolic DosR, and membrane-associated KdpD and cytosolic KdpE [78]. The authors performed a screen using a *Mtb* library and CFP-10 as a bait, which confirmed interactions of CFP-10 with ESAT-6 and identified new interactions of CFP-10 with Rv0686, FtsQ, ClpC1, Pks13, and Rv2240c [78]. Interestingly, the interaction between CFP-10 and mycolic acid condensase Pks13 could not be reproduced in the Y2H system, inferring that this interaction requires a specific mycobacterial environment to be detected [78].

#### 4.1.1. Signaling Pathways

M-PFC was also used to demonstrate interactions between PknH and the response regulator DosR, demonstrating convergence between STPK and TCS signaling in *Mtb* [79]. In combination with *Mtb* proteome microarrays and Y2H approaches, M-PFC was used to further validate interactions between STPK protein interactants and the two selected STPK PknB and PknD [19]. 

#### 4.1.2. Cell Division

In agreement with the BACTH system, M-PFC confirmed interactions between ClpX and FtsZ [57]. M-PFC also confirmed interactions between FtsZ and SepF [80], independently of the screening performed in the Y2H system using FtsZ as a bait, as mentioned above [21]. 

#### 4.1.3. Peptidoglycan Biosynthesis

Mur synthases (MurC-F), which are essential and involved in peptidoglycan biosynthesis in mycobacteria [81], interact with regulatory proteins and proteins involved in cell division, such as PknA and PknB [82]. 

### 4.2. The Split-Protein Sensor (Split-Trp)

**Figure 4:**
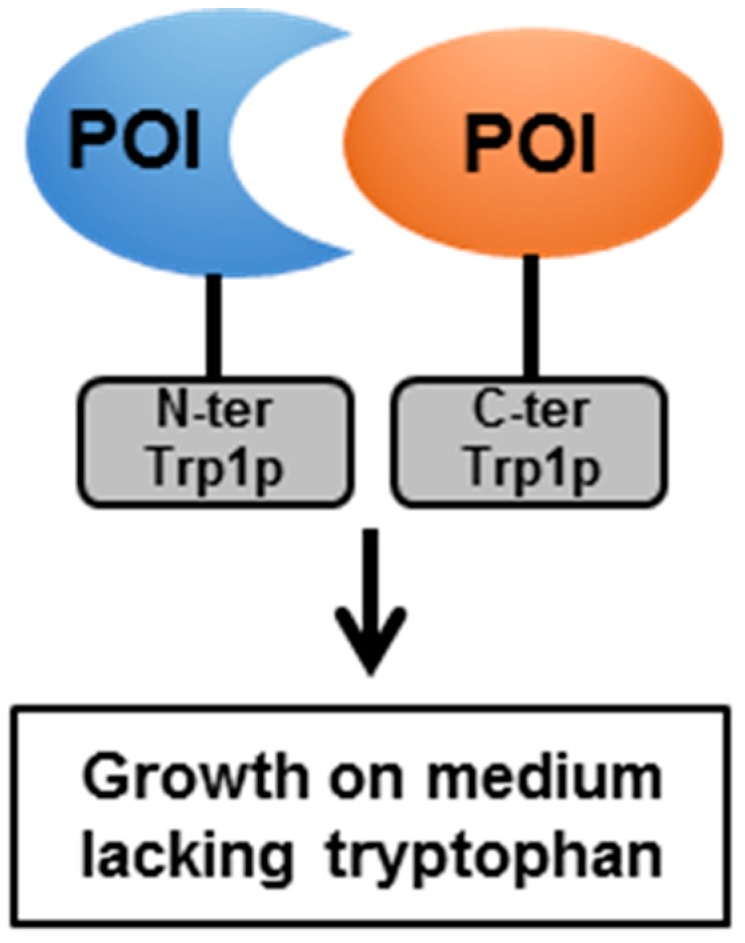
Schematic representation of the split-Trp (or protein fragment complementation assay) technology.

Split-Trp (or protein fragment complementation assay) requires a tryptophan biosynthetic pathway, which is present in mycobacteria. It relies on the reconstitution of an active Trp1p enzyme, only if the POIs interact with each other (Figure 4 and Table 1). This will then allow the tryptophan auxotrophic strain of *M. smegmatis* Δ*hisA* to grow on media without tryptophan [83]. The validity of split-Trp was assessed by confirming interactions between ESAT-6 and CFP-10, and the homodimerization of GlfT1 and RegX3 [83]. In parallel with M-PFC, split-Trp was used to evaluate interactions between PknH and DosR. However, only the phosphorylation-defective form of DosR (T198A/T205A) was able to interact with PknH in this system, suggesting that split-Trp is less sensitive than M-PFC [79].

### 4.3. In Vivo Crosslinking in Live Mycobacteria

**Figure 5:**
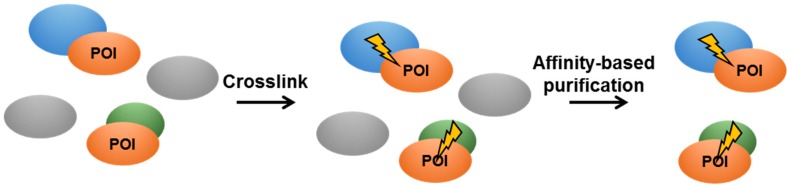
Schematic representation of in vivo crosslinking.

In vivo crosslinking was developed to directly address PPI in a natural environment in order to limit false positive interactions or miss transient interactions (Table 1). It relies on the use of crosslinking agents, such as formaldehyde or (sulfo-)disuccinimidyl suberate, generating covalent adducts of two spatially close proteins (Figure 5). Using formaldehyde as a crosslinking agent, *Mtb* subunit E1 of the pyruvate dehydrogenase complex was shown to interact with nine *M. smegmatis* proteins [84]. Nonetheless, this approach could generate false positives, as naturally biotinylated mycobacterial proteins may interfere with the purification protocol [84].

A more recent approach consists of incorporating the UV-crosslinking unnatural amino acid *p*-benzoylphenylalanine, added to the culture medium, via nonsense suppression in the sequence of the protein under study [85]. Upon UV irradiation of live cells, this allows the formation of a covalent adduct between the studied protein and any interactant, thus capturing physiological interactions in a native environment. This method was applied to the lipoprotein LprG [86], which is involved in cell surface exposure of lipoarabinomannan, the regulation of triacylglycerol levels, phagolysosomal fusion, and *Mtb* virulence [87,88,89]. Among 23 identified interactants, the authors focused on the site-specific interactions of LprG with LppI and LppK, as well as on the physical and functional interactions between LprG and the mycoloyltransferase Ag85A conditioning cell growth and mycolic acid composition [86].

### 4.4. Pros and Cons

Methods developed for use with live mycobacteria are devoted to test direct interactions between potential partners within the mycobacterial environment, in the presence of an adequate cellular organization and the potentially required cofactors or PTM. M-PFC and split-Trp can be used to characterize pairwise interactions, whereas in vivo crosslinking may be useful to demonstrate the existence of protein complexes (Table 1). However, this latter technique is hardly amenable for the development of a high-throughput screening system (Table 1). As distance and orientation between the tested proteins are important, split-Trp may lead to false positive or false negative results, as shown for some ESAT-6 and CFP-10 interactions [83]. Thus, the use of several independent methods in mycobacteria is important in order to eliminate false positive or false negative results.

## 5. Conclusion and Perspectives

All the methods listed above greatly contributed to the understanding of *Mtb* virulence mechanisms by focusing on PPI. However, despite the tremendous amount of data generated by these different technologies, deciphering mycobacterial PPI in terms of multiprotein and dynamic complexes requires more specific and more appropriate systems. In that regard, novel methods, such as in-cell nuclear magnetic resonance (NMR) spectroscopy or the proximity-dependent biotinylation assay, appear to be very promising (Table 1).

In-cell NMR is useful to study the conformation and the dynamics of biological macromolecules (such as protein complexes) under physiological conditions (i.e., within living cells) [90]. For instance, in-cell NRM was used to study the intrinsically disordered mycobacterial protein Pup, a functional analog of ubiquitin [91]. Pup targets mycobacterial proteins for proteasome-mediated degradation, a process that is directly involved in *Mtb* virulence [92]. Pup was studied for its interaction in *E. coli* with the mycobacterial proteasomal ATPase Mpa and with the intact mycobacterial proteasome (Mpa plus *Mtb* proteasome core particle), showing that the proteasome complex had a higher affinity for Pup than Mpa alone [93]. However, the application of in-cell NMR directly in living mycobacteria remains to be tested and further developed.

**Figure 6:**
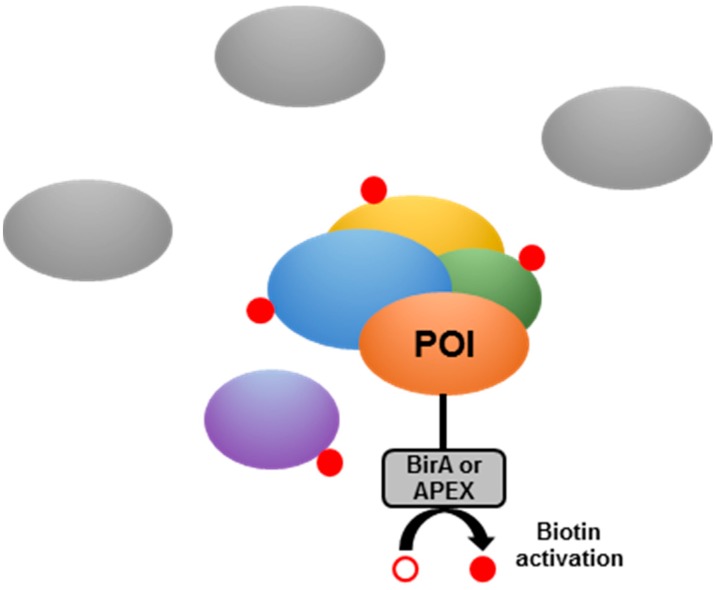
Schematic representation of the proximity-dependent biotinylation assay.

Proximity-dependent biotinylation assays [94] consist of generating a hybrid protein between the POI and a biotin ligase (e.g., a variant of *E. coli* BirA [95] or *A. aeolicus* biotin ligase [96]) or an engineered ascorbic acid peroxidase (e.g., APEX [97,98]) (Figure 6). APEX catalyzes the conversion of its substrate biotin-phenol into short-lived and highly reactive radicals, leading to the covalent attachment of biotin to electron-rich amino acids (such as tyrosines) of proximal proteins [94]. As the technique is directly performed in the organism of interest, whose subcellular structures are kept intact, it greatly minimizes false-positive identifications. The hybrid protein can properly localize, perform its function, and add a biotin residue to all potential partners in spatial proximity (in a 10–20 nm radius). Once the biotin is covalently bound to the proximal proteins, classical lysis methods are not expected to interfere in the process, in contrast to other approaches, such as co-precipitation or tandem affinity purification. The bacterial lysate can then be subjected to purification using streptavidin-based beads or columns. After stringent washes, elution and tryptic digestion, the samples can be subjected to mass spectrometry analysis to detect which biotinylated proteins are enriched in the samples. This method may be particularly suitable for the study of PPI in a natural context, for particular subcellular structures or for proteins involved in specific mycobacterial processes (such as cell wall biosynthesis or virulence mechanisms). Although *Mtb* possesses a biotin synthesis pathway [99] that could interfere with this technique, the use of relevant controls (e.g., a similar production of the POI not fused to the biotin ligase) would allow the identification of a specific subset of enriched biotinylated proteins, representing either direct interactants or spatially-close partners. This technology has not yet been applied to mycobacteria, but may be worthwhile to be tested for the study of *Mtb* PPI.

## Figures and Tables

**Table 1:** Comparison of the different techniques used to study mycobacterial protein–protein interactions (PPI).

Methods	Contact	Membrane Proteins	Nature of the Interaction	Cellular Context	PTM and Cofactors	HTS
Y2H	direct	no	binary	no	no	yes
BACTH	direct	yes	binary	yes/no	yes/no	yes
M-PFC	direct	yes	binary	yes	yes	yes
Split-Trp	direct	yes	binary	yes	yes	yes
Crosslinking	direct	yes	complex	yes	yes	no
In-cell NMR	proximity	yes	complex	yes/no	yes/no	no
Biotinylation	proximity	yes	complex	yes	yes	yes

PTM—post-translational modifications; HTS—high-throughput screening; Y2H—yeast two hybrid; BACTH—bacterial adenylate cyclase-based two-hybrid; M-PFC—mycobacterial protein fragment complementation; NMR—nuclear magnetic resonance.

## References

[1] Bellinzoni M., Wehenkel A.M., Durán R., Alzari P.M. (2019). Novel mechanistic insights into physiological signaling pathways mediated by mycobacterial Ser/Thr protein kinases. Genes Immun..

[2] Melly G., Purdy G.E. (2019). MmpL Proteins in Physiology and Pathogenesis of M. tuberculosis. Microorganisms.

[3] Rameshwaram N.R., Singh P., Ghosh S., Mukhopadhyay S. (2018). Lipid metabolism and intracellular bacterial virulence: Key to next-generation therapeutics. Future Microbiol..

[4] Velasco-García R., Vargas-Martínez R. (2012). The study of protein–protein interactions in bacteria. Can. J. Microbiol..

[5] Schauer K., Stingl K. (2009). ‘Guilty by Association’—Protein-Protein Interactions (PPIs) in Bacterial Pathogens. Genome Dyn..

[6] Forrellad M.A., Klepp L.I., Gioffre A., Sabio y Garcia J., Morbidoni H.R., de la Paz Santangelo M., Cataldi A.A., Bigi F. (2013). Virulence factors of the Mycobacterium tuberculosis complex. Virulence.

[7] Fu H., Miller J., Stagljar I. (2004). Using the Yeast Two-Hybrid System to Identify Interacting Proteins. Protein Protein Interact..

[8] Manganelli R. (2014). Sigma Factors: Key Molecules in Mycobacterium tuberculosis Physiology and Virulence. Microbiol. Spectr..

[9] Hurst-Hess K., Biswas R., Yang Y., Rudra P., Lasek-Nesselquist E., Ghosh P. (2019). Mycobacterial SigA and SigB Cotranscribe Essential Housekeeping Genes during Exponential Growth. mBio.

[10] Steyn A.J.C., Collins D.M., Hondalus M.K., Jacobs W.R., Kawakami R.P., Bloom B.R. (2002). Mycobacterium tuberculosis WhiB3 interacts with RpoV to affect host survival but is dispensable for in vivo growth. Proc. Natl. Acad. Sci. USA.

[11] Garg S., Alam M.S., Bajpai R., Kishan K.R., Agrawal P. (2009). Redox biology of Mycobacterium tuberculosis H37Rv: Protein-protein interaction between GlgB and WhiB1 involves exchange of thiol-disulfide. BMC Biochem..

[12] Lee J.H., Karakousis P.C., Bishai W.R. (2008). Roles of SigB and SigF in the Mycobacterium tuberculosis sigma factor network. J. Bacteriol..

[13] Gebhard S., Hümpel A., McLellan A.D., Cook G.M., McLellan A. (2008). The alternative sigma factor SigF of Mycobacterium smegmatis is required for survival of heat shock, acidic pH and oxidative stress. Microbiology.

[14] Parida B., Douglas T., Nino C., Dhandayuthapani S. (2005). Interactions of anti-sigma factor antagonists of Mycobacterium tuberculosis in the yeast two-hybrid system. Tuberculosis.

[15] Kundu M. (2018). The role of two-component systems in the physiology of Mycobacterium tuberculosis. IUBMB Life.

[16] Steyn A.J.C., Joseph J., Bloom B.R. (2003). Interaction of the sensor module of Mycobacterium tuberculosis H37Rv KdpD with members of the Lpr family. Mol. Microbiol..

[17] Lee H.N., Jung K.E., Ko I.J., Baik H.S., Oh J.I. (2012). Protein-protein interactions between histidine kinases and response regulators of Mycobacterium tuberculosis H37Rv. J. Microbiol..

[18] Prisic S., Husson R.N. (2014). Mycobacterium tuberculosis Serine/Threonine Protein Kinases. Microbiol. Spectr..

[19] Wu F.L., Liu Y., Jiang H.W., Luan Y.Z., Zhang H.N., He X., Xu Z.W., Hou J.L., Ji L.Y., Xie Z. (2017). The Ser/Thr Protein Kinase Protein-Protein Interaction Map of M. tuberculosis. Mol. Cell. Proteom..

[20] Kieser K.J., Rubin E.J. (2014). How sisters grow apart: Mycobacterial growth and division. Nat. Rev. Genet..

[21] Gola S., Munder T., Casonato S., Manganelli R., Vicente M. (2015). The essential role of SepF in mycobacterial division. Mol. Microbiol..

[22] Rosser A., Stover C., Pareek M., Mukamolova G.V. (2017). Resuscitation-promoting factors are important determinants of the pathophysiology inMycobacterium tuberculosisinfection. Crit. Rev. Microbiol..

[23] Hett E.C., Chao M.C., Steyn A.J., Fortune S.M., Deng L.L., Rubin E.J. (2007). A partner for the resuscitation-promoting factors of Mycobacterium tuberculosis. Mol. Microbiol..

[24] Hett E.C., Chao M.C., Rubin E.J. (2010). Interaction and Modulation of Two Antagonistic Cell Wall Enzymes of Mycobacteria. PLoS Pathog..

[25] Quémard A. (2016). New Insights into the Mycolate-Containing Compound Biosynthesis and Transport in Mycobacteria. Trends Microbiol..

[26] Veyron-Churlet R., Guerrini O., Mourey L., Daffé M., Zerbib D., Veyron-Churlet R. (2004). Protein-protein interactions within the Fatty Acid Synthase-II system of Mycobacterium tuberculosis are essential for mycobacterial viability. Mol. Microbiol..

[27] Veyron-Churlet R., Bigot S., Guerrini O., Verdoux S., Malaga W., Daffé M., Zerbib D. (2005). The Biosynthesis of Mycolic Acids in Mycobacterium tuberculosis Relies on Multiple Specialized Elongation Complexes Interconnected by Specific Protein–Protein Interactions. J. Mol. Boil..

[28] Cantaloube S., Veyron-Churlet R., Haddache N., Daffe M., Zerbib D. (2011). The Mycobacterium Tuberculosis FAS-II Dehydratases and Methyltransferases Define the Specificity of the Mycolic Acid Elongation Complexes. PLoS ONE.

[29] Glass L.N., Swapna G., Chavadi S.S., Tufariello J.M., Mi K., Drumm J.E., Lam T.T., Zhu G., Zhan C., Vilcheze C. (2017). Mycobacterium tuberculosis universal stress protein Rv2623 interacts with the putative ATP binding cassette (ABC) transporter Rv1747 to regulate mycobacterial growth. PLoS Pathog..

[30] Guerin M.E., Korduláková J., Alzari P.M., Brennan P.J., Jackson M. (2010). Molecular Basis of Phosphatidyl-myo-inositol Mannoside Biosynthesis and Regulation in Mycobacteria. J. Boil. Chem..

[31] Torrelles J.B., Schlesinger L.S. (2010). Diversity in Mycobacterium tuberculosis mannosylated cell wall determinants impacts adaptation to the host. Tuberculosis.

[32] Renshaw P.S., Lightbody K.L., Veverka V., Muskett F.W., Kelly G., Frenkiel T.A., Gordon S.V., Hewinson R.G., Burke B., Norman J. (2005). Structure and function of the complex formed by the tuberculosis virulence factors CFP-10 and ESAT-6. EMBO J..

[33] Pathak S.K., Basu S., Basu K.K., Banerjee A., Pathak S., Bhattacharyya A., Kaisho T., Kundu M., Basu J. (2007). Direct extracellular interaction between the early secreted antigen ESAT-6 of Mycobacterium tuberculosis and TLR2 inhibits TLR signaling in macrophages. Nat. Immunol..

[34] Feltcher M.E., Sullivan J.T., Braunstein M. (2010). Protein export systems of Mycobacterium tuberculosis: Novel targets for drug development?. Future Microbiol..

[35] Van Winden V.J.C., Houben E.N.G., Braunstein M. (2019). Protein Export into and across the Atypical Diderm Cell Envelope of Mycobacteria. Microbiol. Spectr..

[36] Stanley S.A., Raghavan S., Hwang W.W., Cox J.S. (2003). Acute infection and macrophage subversion by Mycobacterium tuberculosis require a specialized secretion system. Proc. Natl. Acad. Sci. USA.

[37] Champion P.A.D., Stanley S.A., Champion M.M., Brown E.J., Cox J.S. (2006). C-Terminal Signal Sequence Promotes Virulence Factor Secretion in Mycobacterium tuberculosis. Science.

[38] Champion P.A.D., Champion M.M., Manzanillo P., Cox J.S. (2009). ESX-1 Secreted Virulence Factors Are Recognized by Multiple Cytosolic AAA ATPases in Pathogenic Mycobacteria. Mol. Microbiol..

[39] Ribeiro-Guimarães M.L., Pessolani M.C.V. (2007). Comparative genomics of mycobacterial proteases. Microb. Pathog..

[40] Sklar J.G., Makinoshima H., Schneider J.S., Glickman M.S.M. (2010). tuberculosis intramembrane protease Rip1 controls transcription through three anti-sigma factor substrates. Mol. Microbiol..

[41] Makinoshima H., Glickman M.S. (2005). Regulation of Mycobacterium tuberculosis cell envelope composition and virulence by intramembrane proteolysis. Nature.

[42] Schneider J.S., Reddy S.P., Evans H.W., Glickman M.S. (2013). Site-2 protease substrate specificity and coupling in trans by a PDZ-substrate adapter protein. Proc. Natl. Acad. Sci. USA.

[43] Karimova G., Pidoux J., Ullmann A., Ladant D. (1998). A bacterial two-hybrid system based on a reconstituted signal transduction pathway. Proc. Natl. Acad. Sci. USA.

[44] Ladant D., Ullmann A. (1999). Bordatella pertussis adenylate cyclase: A toxin with multiple talents. Trends Microbiol..

[45] Battesti A., Bouveret E. (2012). The bacterial two-hybrid system based on adenylate cyclase reconstitution in Escherichia coli. Methods.

[46] Fontán P.A., Aris V., Alvarez M.E., Ghanny S., Cheng J., Soteropoulos P., Trevani A., Pine R., Smith I. (2008). Mycobacterium tuberculosisSigma Factor E Regulon Modulates the Host Inflammatory Response. J. Infect. Dis..

[47] Casonato S., Provvedi R., Dainese E., Palù G., Manganelli R. (2014). Mycobacterium tuberculosis Requires the ECF Sigma Factor SigE to Arrest Phagosome Maturation. PLoS ONE.

[48] Barik S., Sureka K., Mukherjee P., Basu J., Kundu M. (2010). RseA, the SigE specific anti-sigma factor of Mycobacterium tuberculosis, is inactivated by phosphorylation-dependent ClpC1P2 proteolysis. Mol. Microbiol..

[49] Li Y., Zeng J., He Z.G. (2010). Characterization of a functional C-terminus of the Mycobacterium tuberculosis MtrA responsible for both DNA binding and interaction with its two-component partner protein, MtrB. J. Biochem..

[50] Zahrt T.C., Deretic V. (2000). An Essential Two-Component Signal Transduction System in Mycobacterium tuberculosis. J. Bacteriol..

[51] Fol M., Chauhan A., Nair N.K., Maloney E., Moomey M., Jagannath C., Madiraju M.V.V.S., Rajagopalan M. (2006). Modulation of Mycobacterium tuberculosis proliferation by MtrA, an essential two-component response regulator. Mol. Microbiol..

[52] Gannoun-Zaki L., Alibaud L., Carrère-Kremer S., Kremer L., Blanc-Potard A.B. (2013). Overexpression of the KdpF Membrane Peptide in Mycobacterium bovis BCG Results in Reduced Intramacrophage Growth and Altered Cording Morphology. PLoS ONE.

[53] Chalut C. (2016). MmpL transporter-mediated export of cell-wall associated lipids and siderophores in mycobacteria. Tuberculosis.

[54] Viljoen A., Dubois V., Blaise M., Kremer L., Girard-Misguich F., Girard-Misguich F., Herrmann J.L., Girard-Misguich F., Herrmann J. (2017). The diverse family of MmpL transporters in mycobacteria: From regulation to antimicrobial developments. Mol. Microbiol..

[55] Olvera M.R., Vivès E., Molle V., Blanc-Potard A.B., Gannoun-Zaki L. (2017). Endogenous and Exogenous KdpF Peptide Increases Susceptibility of Mycobacterium bovis BCG to Nitrosative Stress and Reduces Intramacrophage Replication. Front. Microbiol..

[56] Datta P., Dasgupta A., Singh A.K., Mukherjee P., Kundu M., Basu J. (2006). Interaction between FtsW and penicillin-binding protein 3 (PBP3) directs PBP3 to mid-cell, controls cell septation and mediates the formation of a trimeric complex involving FtsZ, FtsW and PBP3 in mycobacteria. Mol. Microbiol..

[57] Dziedzic R., Kiran M., Plocinski P., Ziolkiewicz M., Brzostek A., Moomey M., Vadrevu I.S., Dziadek J., Madiraju M., Rajagopalan M. (2010). Mycobacterium tuberculosis ClpX Interacts with FtsZ and Interferes with FtsZ Assembly. PLoS ONE.

[58] Plocinski P., Ziolkiewicz M., Kiran M., Vadrevu S.I., Nguyen H.B., Hugonnet J., Veckerle C., Arthur M., Dziadek J., Cross T.A. (2011). Characterization of CrgA, a New Partner of the Mycobacterium tuberculosis Peptidoglycan Polymerization Complexes. J. Bacteriol..

[59] Plocinski P., Arora N., Sarva K., Blaszczyk E., Qin H., Das N., Plocinska R., Ziolkiewicz M., Dziadek J., Kiran M. (2012). Mycobacterium tuberculosis CwsA Interacts with CrgA and Wag31, and the CrgA-CwsA Complex Is Involved in Peptidoglycan Synthesis and Cell Shape Determination. J. Bacteriol..

[60] Donovan C., Bramkamp M. (2014). Cell division in Corynebacterineae. Front. Microbiol..

[61] Kruh N.A., Borgaro J.G., Ruzsicska B.P., Xu H., Tonge P.J. (2008). A novel interaction linking the FAS-II and phthiocerol dimycocerosate (PDIM) biosynthetic pathways. J. Boil. Chem..

[62] Cox J.S., Chen B., McNeil M., Jacobs W.R. (1999). Complex lipid determines tissue-specific replication of Mycobacterium tuberculosis in mice. Nature.

[63] Trivedi O.A., Arora P., Vats A., Ansari M.Z., Tickoo R., Sridharan V., Mohanty D., Gokhale R.S. (2005). Dissecting the Mechanism and Assembly of a Complex Virulence Mycobacterial Lipid. Mol. Cell.

[64] Joshi S.A., Ball D.A., Sun M.G., Carlsson F., Watkins B.Y., Aggarwal N., McCracken J.M., Huynh K.K., Brown E.J. (2012). EccA1, a Component of the Mycobacterium marinum ESX-1 Protein Virulence Factor Secretion Pathway, Regulates Mycolic Acid Lipid Synthesis. Chem. Boil..

[65] Cox J.A.G., Abrahams K.A., Alemparte C., Ghidelli-Disse S., Rullas J., Angulo-Barturen I., Singh A., Gurcha S.S., Nataraj V., Bethell S. (2016). THPP target assignment reveals EchA6 as an essential fatty acid shuttle in mycobacteria. Nat. Microbiol..

[66] Larrouy-Maumus G., Škovierová H., Dhouib R., Angala S.K., Zuberogoitia S., Pham H., Villela A.D., Mikušová K., Noguera A., Gilleron M. (2012). A Small Multidrug Resistance-like Transporter Involved in the Arabinosylation of Arabinogalactan and Lipoarabinomannan in Mycobacteria*. J. Boil. Chem..

[67] Belardinelli J.M., Stevens C.M., Li W., Tan Y.Z., Jones V., Mancia F., Zgurskaya H.I., Jackson M. (2019). The MmpL3 interactome reveals a complex crosstalk between cell envelope biosynthesis and cell elongation and division in mycobacteria. Sci. Rep..

[68] Berthet F. (1998). Attenuation of Virulence by Disruption of the Mycobacterium tuberculosis erp Gene. Science.

[69] Klepp L.I., Soria M., Blanco F.C., Bianco M.V., Santangelo M.P., Cataldi A.A., Bigi F. (2009). Identification of two proteins that interact with the Erp virulence factor from Mycobacterium tuberculosis by using the bacterial two-hybrid system. BMC Mol. Boil..

[70] Belon C., Gannoun-Zaki L., Lutfalla G., Kremer L., Blanc-Potard A.B. (2014). Mycobacterium marinum MgtC Plays a Role in Phagocytosis but is Dispensable for Intracellular Multiplication. PLoS ONE.

[71] Belon C., Olvera M.R., Vives E., Kremer L., Gannoun-Zaki L., Blanc-Potard A.B. (2016). Use of the Salmonella MgtR peptide as an antagonist of the Mycobacterium MgtC virulence factor. Future Microbiol..

[72] Alix E., Blanc-Potard A.B. (2008). Peptide-assisted degradation of the Salmonella MgtC virulence factor. EMBO J..

[73] Pethe K., Alonso S., Biet F., Delogu G., Brennan M.J., Locht C., Menozzi F.D. (2001). The heparin-binding haemagglutinin of M. tuberculosis is required for extrapulmonary dissemination. Nature.

[74] Raze D., Verwaerde C., Deloison G., Werkmeister E., Coupin B., Loyens M., Brodin P., Rouanet C., Locht C. (2018). Heparin-Binding Hemagglutinin Adhesin (HBHA) Is Involved in Intracytosolic Lipid Inclusions Formation in Mycobacteria. Front. Microbiol..

[75] Veyron-Churlet R., Dupres V., Saliou J.M., Lafont F., Raze D., Locht C. (2018). Rv0613c/MSMEG_1285 Interacts with HBHA and Mediates Its Proper Cell-Surface Exposure in Mycobacteria. Int. J. Mol. Sci..

[76] Tharad M., Samuchiwal S.K., Bhalla K., Ghosh A., Kumar K., Kumar S., Ranganathan A. (2011). A Three-Hybrid System to Probe In Vivo Protein-Protein Interactions: Application to the Essential Proteins of the RD1 Complex of M. tuberculosis. PLoS ONE.

[77] Wang Y., Cui T., Zhang C., Yang M., Huang Y., Li W., Zhang L., Gao C.H., He Y., Li Y. (2010). Global Protein−Protein Interaction Network in the Human PathogenMycobacterium tuberculosisH37Rv. J. Proteome Res..

[78] Singh A., Mai D., Kumar A., Steyn A.J.C. (2006). Dissecting virulence pathways of Mycobacterium tuberculosis through protein–protein association. Proc. Natl. Acad. Sci. USA.

[79] Chao J.D., Papavinasasundaram K.G., Zheng X., Chávez-Steenbock A., Wang X., Lee G.Q., Av-Gay Y. (2010). Convergence of Ser/Thr and Two-component Signaling to Coordinate Expression of the Dormancy Regulon in Mycobacterium tuberculosis*. J. Boil. Chem..

[80] Gupta S., Banerjee S.K., Chatterjee A., Sharma A.K., Kundu M., Basu J. (2015). Essential protein SepF of mycobacteria interacts with FtsZ and MurG to regulate cell growth and division. Microbiology.

[81] Angala S.K., Belardinelli J.M., Huc-Claustre E., Wheat W.H., Jackson M. (2014). The cell envelope glycoconjugates of Mycobacterium tuberculosis. Crit. Rev. Biochem. Mol. Boil..

[82] Munshi T., Gupta A., Evangelopoulos D., Guzmán J.D., Gibbons S., Keep N.H., Bhakta S. (2013). Characterisation of ATP-Dependent Mur Ligases Involved in the Biogenesis of Cell Wall Peptidoglycan in Mycobacterium tuberculosis. PLoS ONE.

[83] O’Hare H., Juillerat A., Dianišková P., Johnsson K. (2008). A split-protein sensor for studying protein–protein interaction in mycobacteria. J. Microbiol. Methods.

[84] Lougheed K.E., Bennett M.H., Williams H.D. (2014). An in vivo crosslinking system for identifying mycobacterial protein-protein interactions. J. Microbiol. Methods.

[85] Wang F., Robbins S., Guo J., Shen W., Schultz P.G. (2010). Genetic Incorporation of Unnatural Amino Acids into Proteins in Mycobacterium tuberculosis. PLoS ONE.

[86] Touchette M.H., Van Vlack E.R., Bai L., Kim J., Cognetta A.B., Previti M.L., Backus K.M., Martin D.W., Cravatt B.F., Seeliger J.C. (2017). A Screen for Protein-Protein Interactions in Live Mycobacteria Reveals a Functional Link between the Virulence-Associated Lipid Transporter LprG and the Mycolyltransferase Antigen 85A. ACS Infect. Dis..

[87] Shukla S., Richardson E.T., Athman J.J., Shi L., Wearsch P.A., McDonald D., Banaei N., Boom W.H., Jackson M., Harding C.V. (2014). Mycobacterium tuberculosis Lipoprotein LprG Binds Lipoarabinomannan and Determines Its Cell Envelope Localization to Control Phagolysosomal Fusion. PLoS Pathog..

[88] Gaur R.L., Ren K., Blumenthal A., Bhamidi S., Gibbs S., Jackson M., Zare R.N., Ehrt S., Ernst J.D., Banaei N. (2014). LprG-Mediated Surface Expression of Lipoarabinomannan Is Essential for Virulence of Mycobacterium tuberculosis. PLoS Pathog..

[89] Martinot A.J., Farrow M., Bai L., Layre E., Cheng T.Y., Tsai J.H., Iqbal J., Annand J.W., Sullivan Z.A., Hussain M.M. (2016). Mycobacterial Metabolic Syndrome: LprG and Rv1410 Regulate Triacylglyceride Levels, Growth Rate and Virulence in Mycobacterium tuberculosis. PLoS Pathog..

[90] Robinson K.E., Reardon P.N., Spicer L.D. (2012). In-cell NMR spectroscopy in Escherichia coli. Methods Mol. Biol..

[91] Sciolino N., Burz D.S., Shekhtman A. (2019). In-Cell NMR Spectroscopy of Intrinsically Disordered Proteins. Proteomics.

[92] Darwin K.H. (2009). Prokaryotic ubiquitin-like protein (Pup), proteasomes and pathogenesis. Nat. Rev. Genet..

[93] Maldonado A.Y., Burz D.S., Reverdatto S., Shekhtman A. (2013). Fate of Pup inside the Mycobacterium Proteasome Studied by in-Cell NMR. PLoS ONE.

[94] Chen C.L., Perrimon N. (2017). Proximity-dependent labeling methods for proteomic profiling in living cells. Wiley Interdiscip. Rev. Dev. Boil..

[95] Roux K.J., Kim D.I., Raida M., Burke B. (2012). A promiscuous biotin ligase fusion protein identifies proximal and interacting proteins in mammalian cells. J. Cell Boil..

[96] Kim D.I., Jensen S.C., Noble K.A., KC B., Roux K.H., Motamedchaboki K., Roux K.J. (2016). An improved smaller biotin ligase for BioID proximity labeling. Mol. Boil. Cell.

[97] Rhee H.W., Zou P., Udeshi N.D., Martell J.D., Mootha V.K., Carr S.A., Ting A.Y. (2013). Proteomic mapping of mitochondria in living cells via spatially restricted enzymatic tagging. Science.

[98] Lobingier B.T., Hüttenhain R., Eichel K., Miller K.B., Ting A.Y., Von Zastrow M., Krogan N.J. (2017). An Approach to Spatiotemporally Resolve Protein Interaction Networks in Living Cells. Cell.

[99] Salaemae W., Polyak S.W., Booker G.W. (2016). The Role of Biotin in Bacterial Physiology and Virulence: A Novel Antibiotic Target for Mycobacterium tuberculosis. Virulence Mechanisms of Bacterial Pathogens.

